# Circulating cell adhesion molecules in metabolically healthy obesity

**DOI:** 10.1038/s41366-020-00667-4

**Published:** 2020-09-01

**Authors:** Arij Mulhem, Yusef Moulla, Nora Klöting, Thomas Ebert, Anke Tönjes, Mathias Fasshauer, Arne Dietrich, Michael R. Schön, Michael Stumvoll, Volker Richter, Matthias Blüher

**Affiliations:** 1 Medical Department III – Endocrinology, Nephrology, Rheumatology, University of Leipzig Medical Center, Leipzig, Germany; 2 Clinic for Visceral, Transplantation and Thorax and Vascular Surgery, University Hospital Leipzig, Leipzig, Germany; 3 Helmholtz Institute for Metabolic, Obesity and Vascular Research (HI-MAG) of the Helmholtz Zentrum München at the University of Leipzig, Leipzig, Germany; 4 Division of Renal Medicine, Department of Clinical Science, Intervention and Technology, Karolinska Institutet, Stockholm, Sweden; 5 Institute of Nutritional Science, Justus-Liebig-University, Giessen, Germany; 6 Clinic of Visceral Surgery, Städtisches Klinikum Karlsruhe, Karlsruhe, Germany; 7 Institute of Laboratory Medicine, Clinical Chemistry and Molecular Diagnostics, University Hospital Leipzig, Leipzig, Germany

**Keywords:** Obesity, Pre-diabetes

## Abstract

**Background/Objectives:**

People with metabolically healthy obesity (MHO) may still have an increased risk for cardiovascular mortality compared to metabolically healthy lean (MHL) individuals. However, the mechanisms linking obesity to cardiovascular diseases are not entirely understood. We therefore tested the hypothesis that circulating cell adhesion molecules (CAMs) are higher in MHO compared to MHL individuals.

**Subjects/Methods:**

Serum concentrations of soluble intercellular adhesion molecule-1 (sICAM-1), soluble vascular adhesion molecule-1 (sVCAM-1), E-selectin and P-selectin were measured in age- and sex-matched groups of MHL (*n* = 32), MHO categorized into BMI-matched insulin sensitive (IS, *n* = 32) or insulin resistant (IR) obesity (*n* = 32) and people with metabolically unhealthy obesity (MUO, *n* = 32).

**Results:**

Indeed, individuals with MHO have significantly higher sICAM-1, E-selectin, and P-selectin serum concentrations compared to MHL people. However, these CAMs are still significantly lower in IS compared to IR MHO. There was no difference between the groups in sVCAM-1 serum concentrations. Compared to all other groups, circulating adhesion molecules were significantly higher in individuals with MUO.

**Conclusions:**

These findings suggest that obesity-related increased cardiovascular risk is reflected and may be mediated by significantly higher CAMs. The mechanisms causing elevated adhesion molecules even in the absence of overt cardio-metabolic risk factors and whether circulating CAMs could predict cardiovascular events need to be explored.

## Introduction

The concept of metabolically healthy obesity (MHO) derived from clinical observations that a subgroup of people with obesity does not exhibit cardio-metabolic abnormalities [1]. The well documented premature atherosclerosis in people with obesity [2] could be mediated by metabolic (impaired glucose and lipid metabolism), cardiovascular (hypertension, circulating atherogenic factors) or pro-inflammatory abnormalities [1]. Indeed, individuals with MHO have a lower risk to develop cardiovascular diseases (CVDs) compared to people with metabolically unhealthy obesity (MUO) [3, 4]. However, MHO does not seem to represent a benign condition, because people with MHO still have an increased risk for coronary heart disease, cerebrovascular disease, and heart failure compared to metabolically healthy lean (MHL) individuals [4]. Beyond the effects of smoking, elevated LDL-cholesterol, physical activity and fitness, there seem to be obesity-related factors linking fat accumulation or alterations in fat distribution to a higher cardio-metabolic risk even in people with MHO [1, 3].

Factors secreted from adipose tissue may mechanistically link increased fat mass in obesity to endothelial dysfunction and atherosclerotic cardiovascular disease (ASCVD) [5]. In this context, we have shown that MHO is associated with higher adiponectin and lower C-reactive protein (CRP), progranulin, chemerin, fetuin-A, retinol binding protein-4, dipeptidyl peptidase-4 serum concentrations compared to MUO [1, 6]. Among the factors linking obesity to ASCVD, cell adhesion molecules (CAMs) such as endothelial leukocyte adhesion molecule-1 (E-selectin or cluster of differentiation (CD) 62E), platelet (P)-selectin (CD62P), intercellular cell adhesion molecule-1 (ICAM-1; CD54), and vascular cell adhesion molecule-1 (VCAM-1, CD106) may play a mechanistic role. CAMs have been shown to be independently of glucose metabolism parameters associated with obesity [7]. CAMs are glycoproteins expressed on the surface of various cells in response to signals of inflammation and mediate binding with the extracellular matrix [8, 9]. They are involved in migration of leukocytes to inflammatory sites [8]. For example, E-selectin mediates the first adhesion step of monocytes to the vascular wall whereas ICAM-1 and VCAM-1 both mediate adhesion to endothelial cells and transmigration of leukocytes into the subendothelial space [8, 9]. P-selectin mediates the interaction of endothelial cells or platelets with leukocytes [8]. After proteolytic cleavage, the extracellular domain of CAMs is released into the circulation and soluble CAMs (sCAMs) correlate with their cellular surface expression [8]. Serum concentrations of sCAMs are associated with parameters of glycemic control in individuals with type 2 diabetes and prediabetes [10]. Importantly, increased CAMs production particularly in visceral adipose tissue may directly link adiposity to increased ASCVD risk in obesity [11]. We therefore tested the hypothesis that serum CAMs concentrations discriminate age- and sex-matched MHL from MHO either defined as insulin sensitive (IS) or insulin resistant (IR) by euglycemic-hyperinsulinemic clamps.

## Methods

### Subjects

For the purpose of this study, we selected 128 individuals from the Leipzig Obesity Biobank to define age- and sex-matched groups of IS metabolically healthy lean (MHL, *n* = 32), IS (*n* = 32), IR MHO (*n* = 32) and MUO (*n* = 32). IS and IR MHO as well as MUO subgroups were matched for BMI and body fat mass (Table 1). Definition of the MHO subgroups was based on the glucose infusion rate (GIR) during the last 30 min of the steady state in euglycemic-hyperinsulinemic clamps (IS: GIR > 70 μmol/kg/min; IR: GIR < 60 μmol/kg/min) as described [6]. All individuals fulfilled the following inclusion criteria: (1) men or premenopausal women, (2) age >18years, (3) fasting plasma glucose <7.0 mmol/l; (4) HbA1c < 6.0%, (5) stable body weight, defined as the absence of fluctuations of >3% of body weight for ≥3 months before blood tests. In addition, the following exclusion criteria have been defined: (1) medical and family history of type 1 or type 2 diabetes; (2) medical history of hypertension or systolic blood pressure >140 mmHg and diastolic blood pressure >85 mmHg (except for the MUO group); (3) estimated glomerular filtration rate <90 ml/min, (4) any acute or chronic inflammatory disease or symptoms of infection; (5) clinical evidence of either cardiovascular or peripheral artery disease; (6) smoking; (7) LDL-cholesterol >4 mmol/l; (8) any type of malignant disease; (9) thyroid dysfunction; (10) Cushing’s disease or hypercortisolism; (11) alcohol or drug abuse; (12) pregnancy; (13) concomitant medication, except contraceptives. We used the criteria proposed by the Healthy Obese Project to define MHO [12]. BMI was calculated as weight divided by squared height. Waist circumference was measured at the midpoint between the lower ribs and iliac crest. Percentage body fat was measured by bioimpedance analysis. Abdominal visceral and subcutaneous fat areas were calculated using computed tomography or MRI scans at the level of L4–L5. Insulin sensitivity was assessed using the euglycemic-hyperinsulinemic clamp method as described [6]. The study was approved by Ethics committee of the University of Leipzig (approval numbers: 159-12-21052012 and 017-12-23012012) and all subjects gave written informed consent before taking part in the study.

**Table 1:** Characteristics of the study participants.

Study group	Metabolically healthy lean	Metabolically healthy obesity		Metabolically unhealthy obesity
		Insulin sensitive	Insulin resistant	
N (Womenmen)	32 (23/9)	32 (23/9)	32 (23/9)	32 (23/9)
Age (years)	43.7 ± 7.2	42.7 ± 9.4	42.8 ± 6.7	42.7 ± 4.2
Body weight (kg)	66 ± 7	135 ± 21**	130 ± 17**	133 ± 12**
BMI (kg/m²)	23.4 ± 1.5	45.9 ± 6.8**	45.3 ± 4.7**	45.5 ± 3.4**
Body fat (%)	22.6 ± 4.8	44.7 ± 5.6**	45.1 ± 5.4**	44.9 ± 6.1**
Visceral fat area (cm²)	85 ± 86	133 ± 24**	320 ± 78**,^##^	411 ± 119**,^##^,^§^
Subcutaneous fat area (cm²)	119 ± 211	932 ± 124**	926 ± 131**	909 ± 158**
Waist circumference (cm)	84 ± 17	142 ± 13**	139 ± 11**	140 ± 9**
Systolic blood pressure (mmHg)	117 ± 8	121 ± 7	124 ± 4	144 ± 18**, ^##^,^§§^
Diastolic blood pressure (mmHg)	76 ± 6	74 ± 5	74 ± 4	92 ± 11**, ^##^,^§§^
FPG (mmol/l)	5.6 ± 0.6	5.4 ± 0.3	5.7 ± 0.3^##^	5.6 ± 0.5
Impaired fasting glucose (*n*)	4	2	3	4
FPI (pmol/l)	17.6 ± 19.4	28.7 ± 14.1**	106.4 ± 27.1**,^##^	129.9 ± 37.3**,^##^
HbA1c (%)	5.39 ± 0.4	5.33 ± 0.2	5.56 ± 0.3^##^	5.46 ± 0.3
Clamp GIR (µmol/kg/min)	89.5 ± 30	89.3 ± 9.1	29 ± 11**,^##^	35 ± 16**,^##^
Cholesterol (mmol/l)	5.44 ± 0.82	4.84 ± 0.9*	5.11 ± 1.06	5.32 ± 0.94
HDL-Cholesterol (mmol/l)	1.40 ± 0.35	1.39 ± 0.39	1.01 ± 0.27*,^##^	0.83 ± 0.13*, ^##^,^§^
LDL-Cholesterol (mmol/l)	3.24 ± 0.96	2.96 ± 0.88	3.09 ± 0.82	2.85 ± 0.73
Triglycerides (mmol/l)	1.42 ± 0.84	1.23 ± 0.47	1.99 ± 1.18^##^	2.41 ± 1.3^##^,^§^
Free fatty acids (mmol/l)	0.22 ± 0.21	0.22 ± 0.15	0.49 ± 0.19**,^##^	0.55 ± 0.27**,^##^
hsCRP (mg/l)	2.62 ± 2.79	2.61 ± 2.24	3.65 ± 1.48^##^	3.92 ± 0.94^##^
IL-6 (pg/ml)	0.89 ± 1.16	1.31 ± 1.63	2.18 ± 1.69*	2.49 ± 1.05*,^#^
Leukocytes (Gpt/l)	7.8 ± 4.8	7.3 ± 2.4	8.3 ± 4.5	7.9 ± 2.8
ALAT (µkat/l)	0.45 ± 0.17	0.44 ± 0.16	0.51 ± 0.15**	0.54 ± 0.19**
ASAT (µkat/l)	0.39 ± 0.17	0.43 ± 0.14	0.55 ± 0.16^#^	0.59 ± 0.21^#^
GGT (µkat/l)	0.47 ± 0.20	0.44 ± 0.20	0.58 ± 0.22*,^#^	0.53 ± 0.16*,^#^
Adiponectin (µg/ml)	12.1 ± 7.2	6.9 ± 3.4*	3.1 ± 1.5**,^##^	2.7 ± 1.9**,^##^
Leptin (pg/ml)	12 ± 12	41 ± 13**	43 ± 12**	41 ± 9**

Data are means ± SD.

*ALAT* Alanine-Aminotransferase, *ASAT* Aspartate-Aminotransferase, *BMI* body mass index, *FPG* fasting plasma glucose, *FPI* fasting plasma insulin, *GGT* Gamma-Glutamyl Transpeptidase, *HbA1c* glycated hemoglobin, *HDL* high density lipoprotein, *hsCRP* high sensitivity C-reactive protein, *IL-6* Interleukin 6, *LDL* low density lipoprotein.

*p < 0.05; **p < 0.01 for comparisons to metabolically healthy lean control group. ^#^*p* < 0.05; ^##^*p* < 0.01 compared to metabolically healthy obesity group. ^§^*p* < 0.05; ^§§^*p* < 0.01 for comparisons between MUO and IR MHO subgroups.

### Analyses of cell adhesion molecules and other serum parameters

All baseline blood samples were collected between 8 and 10 am after an overnight fast. Serum concentrations of sICAM-1, sVCAM-1, sE-selectin, and sP-selectin were measured by monoclonal antibody-based ELISAs (product numbers DCD540, DVC00, DSLE00, BBE6; R&D Systems, Abington, UK). Plasma insulin was measured with an enzyme immunometric assay for the IMMULITE automated analyzer (Diagnostic Products Corporation, Los Angeles, CA). Serum high-sensitive CRP, adiponectin, interleukin (IL)-6, and leptin were measured as previously described [6].

### Statistical analyses

Data are shown as means ± SD. Statistical analysis was performed using SPSS version 12.0 (Chicago, IL). Group differences were analyzed using Student’s tests. *P* values < 0.05 were considered statistically significant.

## Results

Study participants fulfilling strict criteria of cardio-metabolic health [12] have been selected from a larger biobank (*n* > 5000 donors) to compare serum CAMs concentrations between people with a BMI < 25 kg/m² (MHL) and a BMI ≥ 35 kg/m² (MHO), which do not exhibit main cardio-metabolic risk factors including smoking, high LDL-cholesterol, hyperglycemia, dyslipidemia, hypertension, or any evidence for CVD (Table 1). Study groups were matched for age and sex, but showed differences in parameters of anthropometry, fat distribution, insulin sensitivity, glucose, and lipid metabolism and for the adipokines adiponectin and leptin (Table 1). All subgroups included some individuals with impaired fasting glucose (IFG) (6.1–6.9 mmol/l), but without an imbalanced distribution between the groups (Table 1). To test the potential influence of impaired insulin sensitivity, the MHO group was further categorized into BMI- and body fat mass matched subgroups of IS and IR obesity as described [6]. Compared to the IR MHO subgroup, the MUO subgroup was characterized by significantly higher blood pressure, higher fasting triglyceride, and lower HDL-cholesterol serum concentrations (Table 1). Patients with diabetes were not included into the MUO group.

**Fig. 1:**
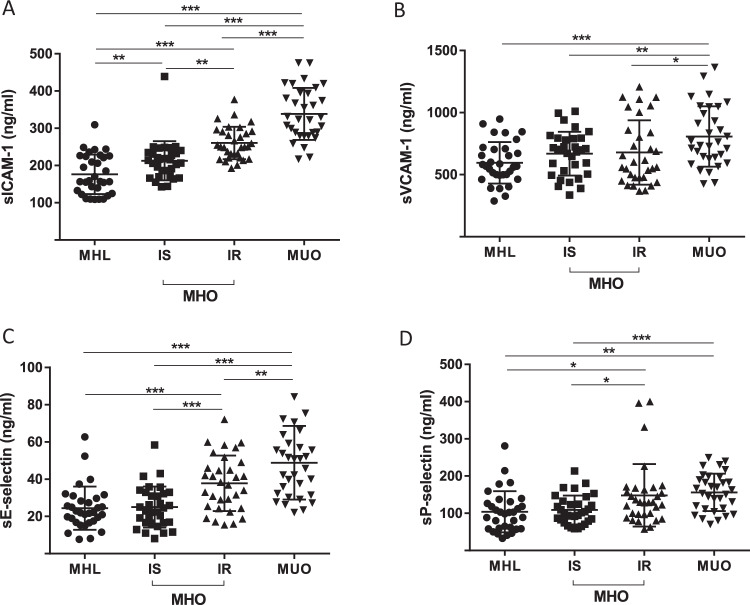
Serum concentrations of soluble ICAM-1, VCAM-1, E-selectin and P-selectin. Comparison between age- and sex-matched groups of metabolically healthy lean (MHL) individuals (BMI ≤ 25 kg/m², *n* = 32), patients with metabolically unhealthy obesity (MUO; BMI ≥ 35 kg/m², *n* = 32), and people with metabolically healthy obesity (MHO; BMI ≥ 35 kg/m²) divided into age-, sex-, and BMI-matched groups of insulin sensitive (IS, *n* = 32) or insulin resistant (IR, *n* = 32) MHO. MHO and MUO were defined by recently reported criteria [12]. Distinction between IS and IR obesity is based on the glucose infusion rate (GIR) in euglycemic-hyperinsulinemic clamp (IS: GIR > 70 μmol/kg/min; IR: GIR < 60 μmol/kg/min) as described in detail previously [6]. **a** sICAM-1, (**b**) sVCAM-1, (**c**) sE-selectin, and (**d**) sP-selectin serum concentrations in the three different groups. *p < 0.05; **p < 0.01; ***p < 0.001. sICAM-1 soluble intercellular adhesion molecule-1, sVCAM-1 soluble vascular cell adhesion molecule-1.

Circulating CAMs concentrations were not different between women and men. We found that sICAM-1, sE-selectin, and sP-selectin serum concentrations are significantly higher in individuals with MHO compared to MHL with additional significant group differences between IS and IR MHO (Fig. 1). The significant differences in circulating CAMs remained significant even after adjusting to main cardio-metabolic risk parameters (blood pressure, LDL-cholesterol, fasting plasma glucose, HbA1c). In contrast, sVCAM-1 concentrations were indistinguishable between the three subgroups (Fig. 1). Serum concentrations of the four adhesion molecules were significantly higher in the MUO subgroup compared to the other subgroups (Fig. 1). We omitted further correlation analyses across the entire cohort to avoid the bias introduced with our group selection criteria.

## Discussion

The key finding of our study is that people with MHO have significantly higher serum concentrations of the CAMs ICAM-1, E-selectin, and P-selectin compared to age- and sex-matched healthy lean individuals. In addition, we identified impaired insulin sensitivity as an additional factor contributing to significantly higher circulating CAMs even within the group of MHO. This observation extends our previous findings that people with IR MHO are characterized by an atherogenic and diabetogenic serum parameter profile that includes lower adiponectin, higher CRP, progranulin, chemerin, fetuin-A, and others compared to people with IS MHO [6]. Higher blood pressure and fasting triglycerides together with lower HDL-cholesterol in the group of MUO is associated with significantly higher circulating adhesion molecules compared to all other groups.

It has been debated whether MHO represents a benign subphenotype of obesity, which may be protected against ASCVD, type 2 diabetes, and other cardio-metabolic diseases [1, 13]. Moreover, individuals with MHO may not benefit from weight loss with regard to their obesity-related cardiovascular and metabolic risk [1, 13]. The concept that MHO is protected against cardio-metabolic diseases has been recently challenged by retrospective analyses of The Health Improvement Network (THIN) database [4]. In this study of 3.5 million men and women from the United Kingdom, CVD events (adjusted for age, BMI, sex, self-reported smoking, and social deprivation) were more frequently found in patients with obesity compared to lean individuals even without overt metabolic abnormalities [4]. It can be argued that higher CVD event risk may result from not systematically studied residual confounding factors such as dietary, behavior, or physical activity factors. In addition, standardized criteria for the definition of MHO are important to translate database findings into potential mechanisms linking obesity to cardio-metabolic disease risk. We therefore tested the hypothesis that circulating CAMs are higher in MHO compared to MHL in a defined human model system of age- and sex-matched groups fulfilling the recently proposed strict diagnostic criteria for metabolic health [12]. Increased CAMs serum concentrations in MHO compared to MHL individuals suggest that higher adipose tissue mass, a known source of increased CAMs production [11], explains the observed differences. However, higher fat mass in MHO compared to MHL did not translate into differences in circulating VCAM-1 in our study. We postulate that the contrasting data for VCAM-1 compared to the other tested CAMs could be caused by specific regulatory factors including adipokines like chemerin. Indeed, chemerin serum concentrations significantly discriminate IS and IR obesity [4]. In this context, it has been shown that chemerin induces ICAM-1 and E-selectin expression in endothelial cells, but not VCAM-1 in vitro [14]. In contrast to sVCAM-1, higher chemerin serum concentrations in children with obesity distinctly correlate with sVCAM-1 and sE-selectin [14].

To further dissect potential mechanisms of increased CAMs serum concentrations, we compared age-, sex-, BMI-, and body fat mass- matched MHO categories of insulin sensitivity and insulin resistance as previously described [6]. Significantly higher ICAM-1, E-selectin, and P-selectin serum concentrations in IR compared to IS MHO suggest that insulin resistance, but also other factors discriminating these two groups such as higher visceral fat mass, adipose tissue inflammation, and alterations in the adipokine secretion pattern play a role in the regulation of CAMs serum concentrations. These data add to our previous findings that hyperinsulinemia and chronic hyperglycemia are related to higher circulating CAMs [10]. There was no subgroup imbalance with regard to the number of individuals with IFG suggesting that those people with IFG-related increased risk for diabetes may not have introduced a bias to the observed group differences in circulating adhesion molecules.

Noteworthy, visceral (but not subcutaneous) adipose tissue has been previously identified as a major source of increased CAMs serum concentrations [11] and may further contribute to the highest circulating CAMs found in the MUO subgroup. In addition, it has been previously shown that monocyte expression of CD11b, one of the counter receptors for CAMs, may be upregulated in patients with obesity depending on adipose tissue immune cell infiltration [15]. Together with our observation that macrophage infiltration into visceral adipose tissue discriminates between IS and IR MHO [6], these data [15] suggest that a low-grade inflammatory response in adipose tissue may link impaired adipose tissue function to both higher circulating CAMs and monocyte CD11b expression.

Taken together, our data suggest that adipose tissue accumulation may be independently of major cardio-metabolic risk factors related to elevated circulating CAMs. Higher sCAMs in people with IR compared to IS MHO further point to additional mechanistic links between adipose tissue dysfunction, systemic insulin resistance, and the potentially atherogenic risk profile even beyond effects of increased fat mass.

Future longitudinal studies are required to test the hypothesis that vascular inflammation related to obesity and impaired adipose tissue function could be early predicted by circulating sCAMs. In addition, the effects of anti-inflammatory pharmacotherapies or behavior interventions on circulating CAMs should be investigated in future trials. Data from our human model system may stimulate studies aiming at identifying the molecular mechanisms underlying the observed associations.
